# Genomic diversity of Areca Palm Velarivirus 1 (APV1) in Areca palm (*Areca catechu*) plantations in Hainan, China

**DOI:** 10.1186/s12864-021-07976-6

**Published:** 2021-10-07

**Authors:** Xianmei Cao, Ruibai Zhao, Hongxing Wang, Huaiwen Zhang, Xue Zhao, Latif Ullah Khan, Xi Huang

**Affiliations:** Hainan Key Laboratory for Sustainable Utilization of Tropical Bioresources, College of Tropical Crops, Hainan University, Haikou, 570228 Hainan People’s Republic of China

**Keywords:** *Areca catechu*, Yellowing leaf disease, APV1, Genetic diversity

## Abstract

**Background:**

Areca palm (*Areca catechu* L.) is an important commercial crop in southeast Asia, but its cultivation is threatened by yellowing leaf disease (YLD). Areca palm velarivirus 1 (APV1) was recently associated with YLD, but little is known regarding its population and genetic diversity. To assess the diversity of YLD, the APV1 genome was sequenced in YLD samples collected from different sites in Hainan.

**Results:**

Twenty new and complete APV1 genomes were identified. The APV1 isolates had highly conserved sequences in seven open reading frames (ORFs; > 95% nucleotide [nt] identity) at the 3′ terminal, but there was diversity (81–87% nt identity) in three ORFs at the 5′ terminal. Phylogenetic analysis divided the APV1 isolates into three phylogroups, with 16 isolates (> 70%) in phylogroup A. Mixed infections with different genotypes in the same tree were identified; this was closely correlated with higher levels of genetic recombination.

**Conclusions:**

Phylogroup A is the most prevalent APV1 genotype in areca palm plantations in Hainan, China. Mixed infection with different genotypes can lead to genomic recombination of APV1. Our data provide a foundation for accurate diagnostics, characterization of etiology, and elucidation of the evolutionary relationships of APV1 populations.

**Supplementary Information:**

The online version contains supplementary material available at 10.1186/s12864-021-07976-6.

## Introduction

Yellowing leaf disease (YLD) was first associated with phytoplasma based on electron microscope observations and PCR amplification of the 16S ribosomal RNA (rRNA) gene in India [1–3], China [4, 5], and Sri Lanka [6, 7]. Areca palm velarivirus 1 (APV1) was first identified in an YLD leaf sample using RNA-sequencing (RNA-Seq) and was associated with YLD in a recent study [8, 9]. APV1 is a member of the genus *Velarivirus* (family Closteroviridae). APV1 has a typical flexuous, filamentous viral particle and a long, positive-sense, single-stranded RNA genome encoding 11 open reading frames (ORFs). ORF1a encodes a large protein with papain-like proteinase (P-PRO), methyltransferase (MET), and helicase (HEL) domains, while ORF1b encodes a protein containing an RNA-dependent RNA polymerase (RdRp) domain; ORF1b is expressed with a frameshift of ORF1a. ORF2 encodes a 4-kDa hydrophobic protein. ORF3 encodes a 70-kDa heat-shock protein 70 homolog (HSP70h) that partially overlaps ORF4, which encodes a 21-kDa polypeptide. ORF5 encodes a 60-kDa protein. ORF6 and ORF7 encode the coat protein (CP) and CP minor (CPm), respectively, whereas ORF8, ORF9, and ORF10 encode 26-, 18- and 19-kDa polypeptides with unknown functions, respectively.

To date, the genomes of only two APV1 isolates have been determined. They showed high genetic diversity in the sequence and genome length. The genome of APV1-WNY (MK956940) is 17,546 nt, whereas that of APV1-HN (KR349464) is 16,080 nt, which was considered incomplete. Both isolates have similar genomic structures, although the 5′ terminal sequences show significant variation with only 83% nt identity [8, 9]. The lack of genomic sequences has hampered evaluation of the genetic diversity, pathogenicity, diagnostics, epidemiological characteristics, and evolutionary relationships of APV1. High-throughput sequencing (HTS) is a rapid, efficient, cost-effective platform for analyzing the genetic diversity of plant viruses and viroid genomes [10]. Here, HTS combined with RT-PCR amplification was used to detect YLD samples from different collection sites in Hainan Province. Twenty complete APV1 genome sequences were obtained. The sequences described here highlight the genetic diversity and phylogroups in the APV1 population. This has clear implications for accurate diagnostics, providing a foundation for elucidating the epidemiological characteristics and evolutionary relationships of APV1 populations.

## Materials and methods

### Plant sample collection and RNA extraction

Fifteen areca palm leaf samples with typical YLD symptoms were collected in the cities of Wanning and Qionghai, and in Lingshui, Tunchang, Baoting, and Ledong Counties in Hainan Province, China (Fig. S1). The samples were stored at − 80 °C until RNA isolation.

### RNA-Seq and de novo assembly

YLD leaves samples were ground separately in liquid nitrogen. Total RNA from each sample was isolated using Tiangen plant RNA isolation kit according to the manufacturer’s instructions (Tiangen Biotech, Beijing, China). RNA-Seq and de novo assembly were performed separately for each YLD sample, as described previously [9]. After annotation, the APV1 unigenes were selected. Gaps and terminals were amplified by RT-PCR using PrimeSTAR® GXL DNA Polymerase (TaKaRa, Dalian, China). The PCR products were incubated with *Taq* polymerase at 72 °C for 10 min and then ligated into the pMD-19 T vector (TaKaRa). Three independent positive clones of each fragment were subject to Sanger sequencing (Sangon Biotech, Guangzhou). Overlapping sequences were assembled into complete genomes using SeqMan Pro 7.1.0 (DNAStar Inc., Madison, WI, USA).

### Phylogenetic and sequence analysis of APV1

For phylogenetic analysis, all full-length genome sequences obtained in this study were used, together with two APV1 sequences available in GenBank. The sequences were aligned using ClustalW with the default parameters and a phylogenetic tree was constructed using the neighbor-joining method with MEGA 7.0.

### Recombination analysis

Recombination events were analyzed with the Recombination Detection Program (RDP v.4.95) under the default conditions, using an alignment of complete APV1 genome sequences constructed with MAFFT v7 [11].

### Evaluation of primers for the detection of APV1

To identify a primer pair with a broad detection range, all reported primers for detecting the virus were compared in silico with APV1 complete genome sequences.

## Results

### RNA-Seq and APV1 genome assembly

Fifteen separate YLD samples were subjected to RNA extraction and RNA-Seq. Following de novo assembly of the sequence reads using Trinity, with an overlap length of k-mer = 25 [12], BlastN and BlastX analyses (cut-off value = 10^− 3^) revealed APV1 unigenes in each YLD sample. Thirteen assembled APV1 unigenes were longer than 17,000 nt, which almost cover the complete APV1 genome sequences (17,546 nt) (Table 1). The full-genome sequences were determined by RT-PCR amplification of the gaps and terminal ends: 20 new complete genome sequences of APV1 isolates were identified. Interestingly, more than one different APV1 isolate was identified in each of three YLD samples (WNXL1, LSGP1, and WNCF1). The APV1 genome sequences were deposited in GenBank under accession numbers MW316004–MW316024. Previously, we used two YLD samples (WNY and BTY) for RNA-Seq [9], but only the genome of WNY isolate was determined. Here, the complete genome of the BTY isolate was determined through RT-PCR and rapid amplification of cDNA ends (RACE). Both isolates have the same 17,564 nt genome length, and identical 5′-UTR (48 nt) and 3′-UTR (225 nt) lengths. Furthermore, both isolates share a highly conserved 3′-UTR (99.6% nt identity) and seven ORFs (ORF4 to ORF10) at the 3′ terminal (97.6% nt identities). However, notable sequence variation was observed at ORF1a (81.0% nt and 81.6% aa identities), suggesting that it belongs to different genotypes.

**Table 1:** Summary of the RNA-Seq, de novo assembly, and APV1 isolate data of areca palm YLD samples

Sample	Clean reads	Clean bases	Q30 (%)	GC (%)	Unigenes	APV1 unigenes	Longest APV1 unigenes	APV1 isolates identified
Control	112,977,362	16.95G	93.36	43.5	322,485	0	0	
BTMT1	109,014,442	16.35G	93.64	44.25	375,447	9	17,448 nt	BTMT1
BTZL1	111,153,834	16.67G	93.48	44.3	307,293	8	17,440 nt	BTZL1
LDTT1	114,521,988	17.18G	93.57	43.84	409,421	9	17,515 nt	LDTT1
LDTT2	95,489,440	14.32G	93.3	43.96	360,780	9	17,530 nt	LDTT2
LSGP1	92,771,398	13.92G	93.44	44.37	273,553	26	9318 nt	LSGP1–1; LSGP1–2; LSGP1–3
LSGP2	97,687,560	14.65G	93.52	43.72	290,986	8	17,482 nt	LSGP2
QHDH1	89,537,286	13.43G	94.09	44.53	324,586	13	17,435 nt	QHDH1
QHDH4	80,108,502	12.02G	93.64	44.03	260,972	13	17,438 nt	QHDH4
QHZY1	87,295,496	13.09G	93.78	45.27	322,034	13	17,481 nt	QHZY1
QHZY2	95,210,422	14.28G	93.76	43.87	318,040	12	17,438 nt	QHZY2
TCFM1	115,107,346	17.27G	93.56	44.96	363,079	9	17,501 nt	TCFM1
WNCF1	98,937,520	14.84G	93.25	43.77	345,757	31	8866 nt	WNCF1–1, 1–2, and 1–3
WNLG1	122,943,318	18.44G	93.42	44.19	338,253	8	17,437 nt	WNLG1
WNSG1	82,280,618	12.34G	93.46	44.38	312,624	6	17,434 nt	WNSG1
WNXL1	98,334,604	14.75G	93.14	43.7	394,940	31	17,482 nt	WNXL1–1, WNXL1–2

### Genome organization and sequence similarities of the new APV1 isolates

**Fig. 1:**
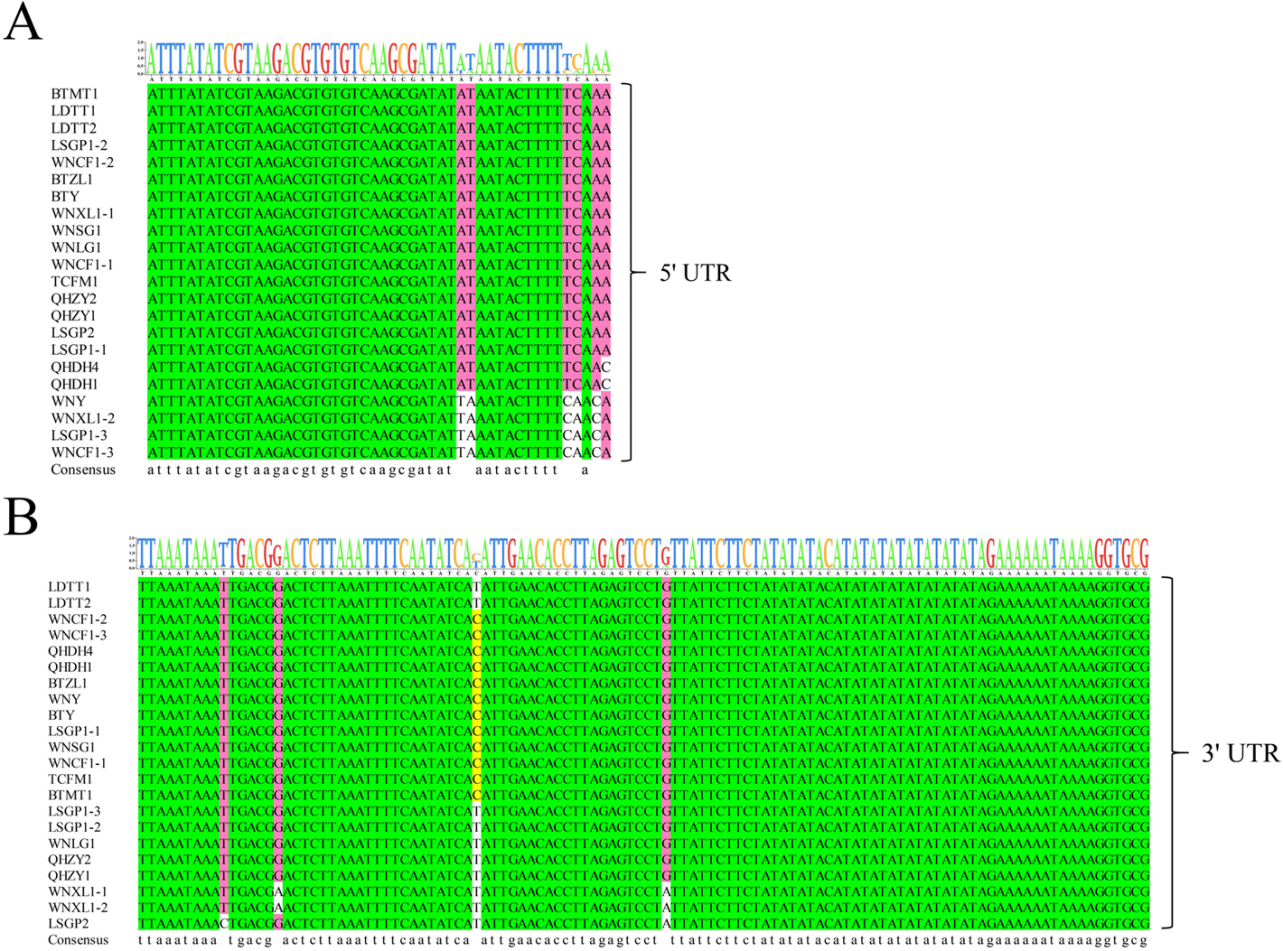
Alignment of the 5′ untranslated region (UTR) (48-nt) and part of the 3′-UTR (113-nt)

All the newly identified APV1 isolates have 11 ORFs and share identical genome organization with the reported WNY and APV1-hn isolates. Sixteen new isolates shared 90% nt genome identity with isolate APV1-WNY, with the greatest sequence variation seen in three ORFs at the 5′ terminal (ORF1a, ORF1b, and ORF2), whereas the eight ORFs at the 3′ terminal (ORF3 to ORF10) had high nt and amino acid (aa) similarity (Table 2). Despite the significant nt variation (87% nt identity), the aa sequences of ORF1b (encoding RdRp) were conserved (≥ 94% aa identity) between WNY and these isolates. Three isolates (LSGP1–3, WNCF1–3, and WNXL1–2) had the highest entire genome nt similarity (98% genomic nt identity) with isolate WNY, whereas isolates LSGP1–2 and WNCF1–2 had 94 and 95% identity, respectively. These isolates were collected from two adjacent regions, Wanning City or Lingshui County. Interestingly, mixed infections with different APV1 genotypes were identified in samples LSGP1, WNCF1, and WNXL1 (Table 2). No indels or insert polymorphisms were found in any isolate. The 5′- and 3′-UTR nts were relatively conserved among the APV1 isolates (Fig. 1). Additionally, to identify a primer pair with a broader detection range, a primer set CPnew based on APV1 CP consensus sequences (with no SNP in 23 known APV1 isolates) was designed for diagnosing various APV1 isolates. (CPnew-F: 5′-ATCGCTAAATATTATGGATAGACTT-3′; CPnew-R: 5′-TATTCAGAAGCATAAGATTGTGACA-3′). All the previously reported primers for detecting the virus were compared in silico with 20 APV1 complete genome sequences [9], the SNPs of each primers were shown in Figure S2. Finally, Twenty areca palm leafs samples were collected from different plantation areas of Hainan and RT-PCR was used to detect the presence of APV1. The detection rates are highly dependent on the SNP number. The primer sets with no SNP show higher detection rates and primer set with most SNP (YLDV1 and YLDV6) show lower detection efficiency (Figure S3), indicating that the primers based on consensus sequences have a broader detection range.

**Table 2:** Nucleotide (amino acid) identity (%) between APV1-WNY and the new APV1 isolates

Isolates	Accession No	Full genome	ORF1a (PRO, MET, HEL)	ORF1b (RdRp)	ORF2 (P4)	ORF3 (HSP70)	ORF4 (P21)	ORF5 (P60)	ORF6 (CP)	ORF7 (CPm)	ORF8 (P26)	ORF9 (P18)	ORF10 (P19)
APV1-HN	KR349464	–	–	87 (94)	86 (78)	96 (98)	98 (98)	97 (97)	99 (99)	97 (95)	98 (99)	98 (95)	–
BTMT1	MW316004	90	81 (82)	87 (94)	86 (75)	96 (98)	98 (98)	98 (98)	99 (99)	97 (97)	98 (99)	98 (95)	97 (97)
BTY	MW316018	90	81 (82)	87 (94)	86 (75)	96 (98)	98 (98)	98 (98)	99 (99)	97 (95)	98 (99)	98 (95)	97 (97)
BTZL1	MW316019	90	81 (82)	87 (94)	86 (75)	96 (98)	98 (98)	98 (98)	99 (99)	97 (95)	98 (99)	98 (95)	97 (97)
LDTT1	MW316005	90	81 (82)	87 (94)	85 (75)	96 (98)	98 (98)	97 (98)	98 (99)	97 (96)	99 (99)	97 (95)	98 (97)
LDTT2	MW316006	90	81 (82)	87 (94)	85 (75)	96 (98)	98 (98)	97 (98)	98 (98)	97 (96)	99 (99)	97 (95)	98 (98)
LSGP1–1	MW316007	90	81 (82)	87 (94)	86 (75)	96 (98)	98 (98)	98 (98)	98 (99)	97 (95)	99 (100)	99 (99)	99 (99)
LSGP1–2	MW316020	94	91 (90)	92 (98)	86 (75)	97 (98)	98 (98)	98 (98)	99 (99)	97 (95)	97 (98)	98 (95)	99 (99)
LSGP1–3	MW316023	98	100 (99)	92 (98)	86 (75)	97 (98)	98 (98)	98 (98)	99 (99)	97 (95)	97 (99)	98 (95)	99 (99)
LSGP2	MW316008	90	81 (82)	87 (94)	84 (72)	97 (99)	98 (98)	98 (98)	98 (99)	97 (96)	99 (100)	97 (95)	98 (97)
QHDH1	MW316009	90	81 (82)	87 (94)	85 (75)	96 (98)	98 (98)	98 (98)	98 (98)	97 (96)	98 (100)	98 (96)	98 (97)
QHDH4	MW316010	90	81 (82)	87 (94)	85 (75)	96 (98)	98 (98)	98 (98)	99 (99)	97 (96)	98 (100)	98 (96)	98 (97)
QHZY1	MW316011	90	81 (82)	87 (94)	84 (72)	96 (99)	98 (98)	97 (97)	98 (99)	97 (96)	99 (100)	97 (94)	98 (97)
QHZY2	MW316012	90	81 (82)	87 (94)	85 (75)	96 (99)	98 (98)	97 (97)	98 (99)	97 (96)	99 (100)	97 (94)	98 (97)
TCFM1	MW316013	90	81 (82)	87 (94)	85 (75)	96 (99)	98 (99)	98 (98)	98 (99)	98 (98)	99 (100)	99 (99)	98 (96)
WNCF1–1	MW316014	90	81 (82)	87 (94)	84 (72)	97 (99)	98 (99)	98 (98)	99 (99)	97 (96)	99 (100)	99 (99)	99 (99)
WNCF1–2	MW316021	95	91 (90)	92 (97)	86 (72)	99 (99)	98 (100)	99 (100)	98 (99)	98 (96)	98 (99)	99 (99)	99 (99)
WNCF1–3	MW316024	98	99 (99)	92 (97)	86 (72)	99 (99)	99 (100)	99 (100)	98 (99)	98 (96)	99 (100)	99 (99)	99 (99)
WNLG1	MW316015	90	81 (82)	87 (95)	85 (75)	96 (99)	98 (98)	97 (97)	98 (98)	97 (96)	99 (100)	98 (95)	98 (97)
WNSG1	MW316016	90	81 (82)	87 (94)	85 (75)	96 (99)	98 (98)	97 (97)	98 (98)	97 (96)	99 (100)	98 (95)	98 (97)
WNXL1–1	MW316017	90	81 (82)	87 (94)	84 (72)	97 (99)	98 (98)	98 (98)	98 (99)	97 (96)	99 (100)	98 (95)	98 (98)
WNXL1–2	MW316022	98	99 (99)	99 (100)	88 (78)	97 (99)	98 (98)	98 (98)	98 (98)	97 (96)	99 (100)	98 (95)	98 (98)

### Phylogenetic analysis of APV1 isolates

**Fig. 2:**
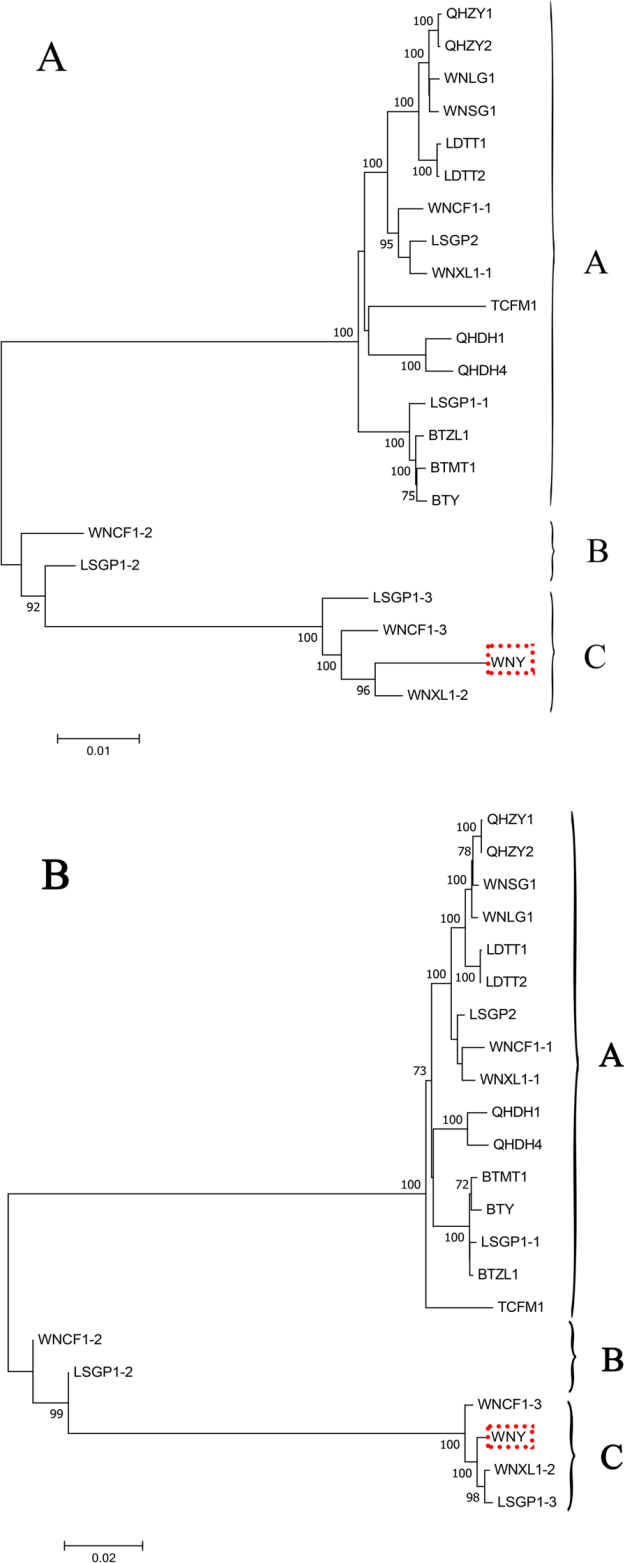
Phylogenetic analyses of APV1 isolates. **A** Phylogenetic tree based on the complete genome sequence of APV1 isolates. **B** Phylogenetic tree based on ORF1a nucleotide sequences of APV1 isolates. Isolate WNY is in the red box. Bootstrap values over 70% are shown

To reveal the evolutionary relationships of the APV1 isolates, phylogenetic trees were constructed using either the full genome or ORF1a nt sequence (Fig. 2A). In the phylogenetic tree based on the full-genome nt sequences, the isolates clustered into three phylogroups. Sixteen APV1 isolates with evident sequence variation from isolate WNY were grouped in phylogroup A; the isolates with the highest sequence similarities to isolate WNY (LSGP1–3, WNCF1–3, WNXL1–2, and WNY) formed phylogroup C, and the remaining isolates (LSGP1–2 and WNCF1–2) formed phylogroup B. Three isolates identified from sample LSGP1 (LSGP1–1, LSGP1–2, and LSGP1–3) and three from sample WNCF1 (WNCF1–1, WNCF1–2, and WNCF1–3), were separated into three different phylogroups. Two isolates from sample WNXL1 (WNXL1–1 and WNXL1–2) were in phylogroups A and C, respectively. Phylogroup B consisted of two isolates (LSGP1–2 and WNCF1–2) located between phylogroups A and C, suggesting that the phylogroup B is a genotype derived from recombination between phylogroups A and C (Fig. 2A). Because ORF1a shows most significant sequence variation among the APV1 isolates, nt sequences of ORF1a were selected for phylograph clustering. The phylogenetic tree based on ORF1a resulted in similar phylogroup clustering (Fig. 2B), indicating that the sequence variation in ORF1a represents the genetic diversity among the APV1 isolates.

### Recombination analysis

**Fig. 3:**
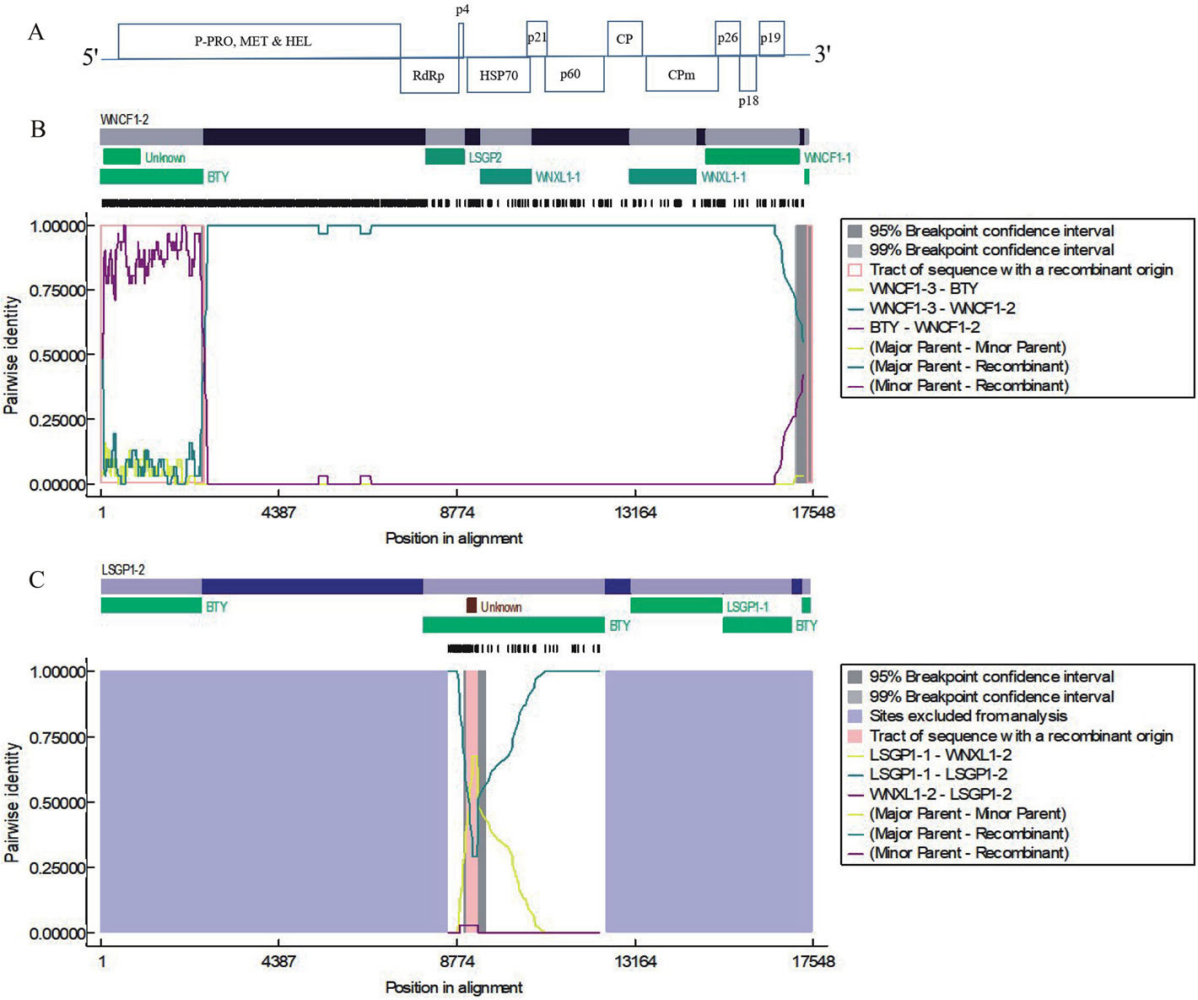
Recombination events predicted by RDP4. **A** Schematic of the APV1 genome organization. The positions of identified recombinants are shown along with the names of the parental sequences. The individual genome of the APV1 isolate is indicated by different colored bars. The identified recombination events, including WNCF1–2 (**B**) and LSGP1–2 (**C**), were rechecked using RDP4 and plotted with pairwise identity information

To investigate the evolutionary relationships of the APV1 populations, recombination analysis was performed with RDP4 [11] using different algorithms (RDP, GENECONV, BootScan, MaxChi, Chimaera, and 3Seq) on an alignment of all full-length APV1 genome sequences. RDP4 revealed 20 recombination events from 22 viral genomes, of which, 15 recombination events was detected in the isolates from mixed infection samples (LSGP1, WNCF1, and WNXL1) (Table 3), strongly suggesting that co-infection with different APV1 genotypes leads to genetic recombination. On examining the recombination events in isolates WNCF1–2 and LSGP1–2 (clustered into phylogroup B), WNCF1–1 and BTY were revealed as the parental isolates of WNCF1–2, while BTY and LSGP1–1 were the parental isolates of LSGP1–2, indicating that the major combination source come from co-infected APV1 isolates (Fig. 3), suggesting that sequence diversity among different APV1 phylogroups contributes to genetic recombination. Furthermore, when we marked the collected sites and APV1 isolates on map, we can clearly see that the major recombination correlated isolates, such as BTY, WNCF1–2, WNXL1–2 and LSGP1–2 variants are geographical neighbor (Fig. S1), suggesting a correlation between geographical distance and recombination events.

**Table 3:** Recombination events predicted by RDP4 in the full genomes of the 22 APV1 isolates

Event No.	Recombination	Major parent	Minor parent	Detection methods						
				R	G	B	M	C	S	T
1	LSGP1–1	BTZL1	Unknown (WNLG1)	+	+	–	–	–	–	–
2	LSGP1–2	LSGP1–1	Unknown (WNXL1–2)	–	+	–	+	+	–	+
3	LSGP1–3	WNY	LSGP1–1	–	+	–	–	–	+	+
4	LSGP1–3	Unknown (TCFM1)	BTY	+	+	–	–	–	–	–
5	LSGP1–3	WNY	BTY	+	+	–	+	+	+	+
6	LSGP2	WNXL1–1	QHZY1	+	+	–	+	+	–	–
7	QHDH4	QHZY1	QHDH1	–	–	–	+	+	–	–
8	QHZY1	LSGP2	Unknown (QHDH1)	–	–	–	+	+	–	–
9	TCFM1	BTZL1	WNY	+	–	–	+	+	–	–
10	WNCF1–1	WNXL1–1	Unknown (QHZY1)	–	–	–	+	–	–	+
11	WNCF1–2	WNCF1–3	BTY	+	+	–	+	+	+	+
12	WNCF1–3	WNY	LSGP2	+	+	–	+	+	+	+
13	WNCF1–3	Unknown (WNLG1)	WNCF1–1	+	+	–	+	+	–	–
14	WNCF1–3	WNY	WNXL1–1	+	+	–	+	+	–	–
15	WNCF1–3	WNY	WNXL1–1	+	+	–	+	–	+	–
16	WNSG1	LDTT1	BTZL1	+	–	–	+	+	–	–
17	WNXL1–2	WNY	WNXL1–1	+	–	–	–	–	+	–
18	WNXL1–2	Unknown (TCFM1)	WNXL1–1	+	+	–	–	–	+	–
19	WNXL1–2	Unknown (QHDH1)	WNXL1–1	+	+	–	–	–	–	–
20	WNXL1–2	WNY	LSGP2	+	+	–	+	+	+	+

## Discussion

Little is known regarding the population and genetic diversity of APV1. HTS technology enables the discovery and identification of unknown viral genome sequences [13, 14]. Here, HTS was used to investigate the genetic diversity of APV1 in areca palm plantations in Hainan, China. In total, 20 new APV1 complete genomes were identified. From 15 YLD samples collected in Hainan, 12 were infected by a single APV1 isolate and 3 showed mixed infection by two or three different APV1 isolates. Co-infection by different viral genotypes within the same host have been reported in many crops and viruses [10, 13, 15, 16]. In this study, recombination analysis first revealed a correlation between a high recombination rate and mixed infections of APV1 isolates (Fig. 2 and Table 3). Furthermore, the isolates of phylogroup B might have resulted from genetic recombination of co-infecting isolates from phylogroups A and C (Figs. 2A and 3). These results provide very important evidences of the evolutionary relationships of APV1 populations.

The classification of viral species is usually based on molecular and biological characteristics, as well as phylogenetic relationships. The sequence divergence criterion for species demarcation within the family Closteroviridae was increased from 10 to 25% for phylogenetically informative proteins (e.g., RdRp, HSP70h, or CP) [17]. Based on this criterion, all of the isolates identified herein belong to species APV1. The isolates clustered into three different phylogenetic groups based on complete genome sequences. The genetic variation that differentiated the three APV1 phylogroups was concentrated in three 5′-terminal ORFs, whereas the eight 3′-terminal ORFs had high sequence similarity. ORF1a shows most significant sequence variation among the APV1 isolates (Table 2). The phylogenetic tree based on ORF1a resulted in similar phylogroup clustering (Fig. 2B), variation of ORF1a is the representative genetic diversity of APV1 isolates. Instead of the entire genome, ORF1a could be selected for the target of APV1 genetic diversity in the future study, which could save time and money.

Through phylogenetic analysis, phylogroup A was found to be the most prevalent genotype in areca palm plantations in Hainan, China, but the reasons are so far less known. Characterization of the association between epidemiological characteristics and genotypes, and identification of the interaction between the different genotypes and insect vectors might be required for the characterization of etiology and propagation of the disease.

Sequence comparisons of all available APV1 isolates showed high conservation in the 5′-UTR, and in most of the 3′-UTR. While there were several single nt polymorphisms, no indel polymorphisms were identified in either UTR region for any APV1 isolate. The 3′-UTR of positive-strand RNA viruses generally contains regulatory sequences essential for both host protein binding and viral multiplication [18, 19]. Sequence conservation in the 3′-UTR might be required for efficient APV1 viral replication. Most positive-strand RNA plant viruses lack the 5′-cap or the poly(A)-tail, which act synergistically to stimulate the canonical translation of cellular mRNAs. However, they have RNA elements in the 5′- or 3′-UTRs, which are required for cap-independent translation [20]. Citrus tristeza virus (CTV) is a well-studied member of Closteroviridae. Many CTV isolates share similar 5′-UTR structures with two long stem loops, which are the primary determinants of recognition and the initiation of replication [21]. APV1 5′-UTR is shorter than the CTV 5′-UTR; the secondary structures are also very different, suggesting that a different mechanism regulates viral replication in APV1.

Consensus among the eight 3′-terminal ORFs enabled us to design PCR primers for diagnosing various APV1 isolates. The nt and aa sequences of ORF6 (CP), ORF7 (CPm), and ORF8 (P26) are very similar across the isolates, and constitute optimal targets for engineering virus-resistant plants, such as by over-expression of RNA interference (RNAi), artificial microRNAs (amiRNAs), synthetic trans-acting small interfering RNAs (syn-tasiRNAs) [22–25], CRISPR [26, 27], and virus-based vaccines [28, 29]. However, these methods all depend on *Agrobacterium* or virus-mediated transformation, neither of which is available for areca palm.

Future research should examine the epidemiological characteristics of different APV1 genotypes, which might help to identify a mild isolate. Pre-inoculation with a mild isolate has successfully induced cross-protection against virulent isolates of many plant viruses [30, 31]. We also plan to construct a wild-type infectious clone, and to create a mild infectious clone by attenuating the APV1 genome.

## Supplementary Information

**Additional file 1.**

## Data Availability

The complete APV1 genome sequences were deposited in GenBank with respective accession numbers MW316004–MW316024 and the datasets used and/or analysed during the current study available from the corresponding author on reasonable request.
