# Supplementary material for: Genomic diversity of Areca Palm Velarivirus 1 (APV1) in Areca palm (Areca catechu) plantations in Hainan, China
Source: BMC Genomics. 2021 Oct 7;22:725. doi: 10.1186/s12864-021-07976-6 (PMC8499421; doi:10.1186/s12864-021-07976-6)
Supplement: Supplementary file 1 — Additional file 1. [file 12864_2021_7976_MOESM1_ESM.pdf]

Genomic diversity of Areca Palm Velarivirus 1 (APV1) in Areca palm (*Areca catechu*) plantations in Hainan, China

Xianmei Cao, Ruibai Zhao, Hongxing Wang, Huaiwen Zhang, Xue Zhao, Latif Ullah Khan, Xi Huang*

Hainan Key Laboratory for Sustainable Utilization of Tropical Bioresources, College of Tropical Crops, Hainan University, Haikou 570228, Hainan, P. R. China

*Author for correspondence

Email: xihuang@hainanu.edu.cn

Fax: +86-898-66258650

Tel: +86-13034998110

Supplemental materials

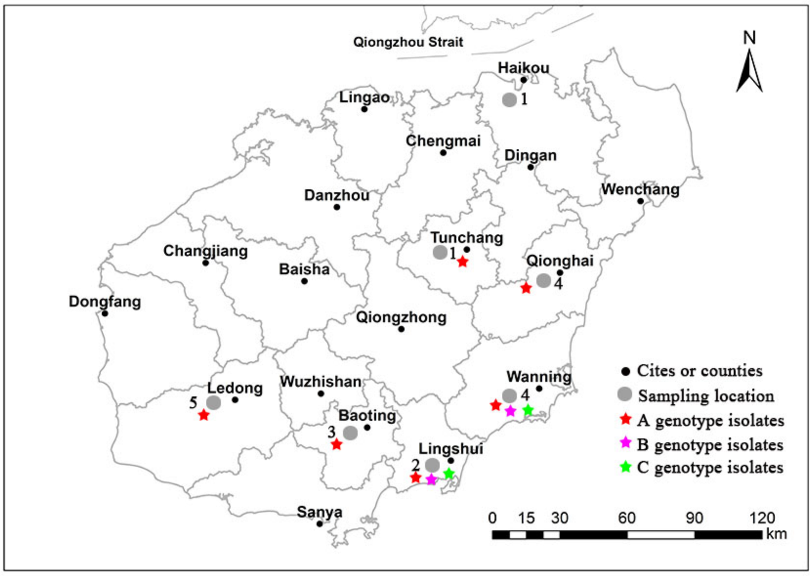

Figure S1. Geographical locations of the areca palm samples collection and distributions of different APV1 isolates in Hainan. The first two letters of APV1 isolates names indicates the abbreviations of the Cities or Counties of samples collection. BT: Baoting; QH: Qionghai; LD: Ledong; LS: Lingshui; TC: Tunchang; WN: Wanning. The following two letters indicate the towns or villages of the samples collection sites in this study. BTY and WNY were isolates identified in samples collected from Baoting and Wanning in previous report, respectively (Wang et al., 2020). The numbers indicate the APV1 isolates identified.

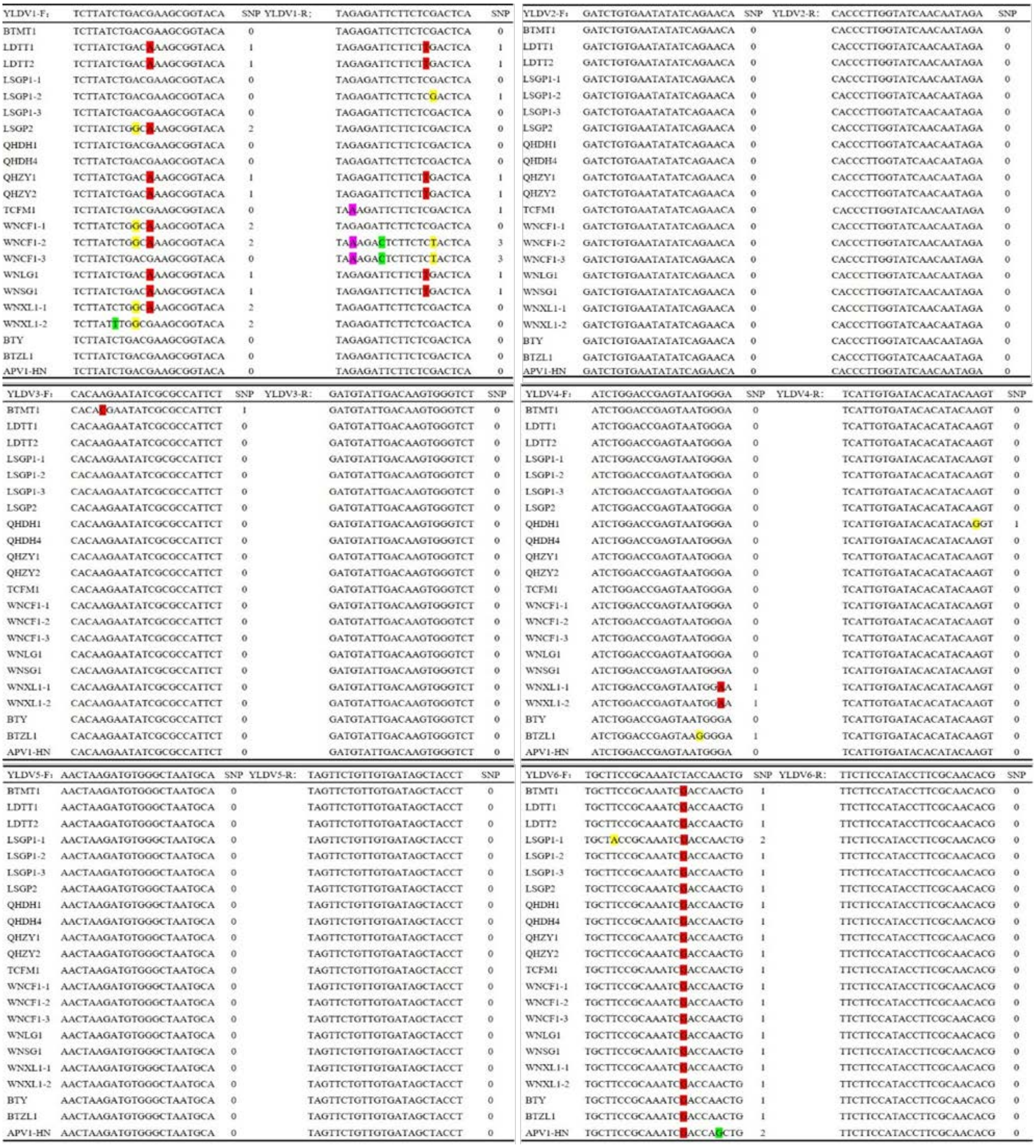

Figure S2. Single nucleotide polymorphism (SNP) of the primers designed for APV1 detection. The position and number of mismatches of the primer with the sequences of 20 APV1 isolates. Colors highlight the different mismatched bases.

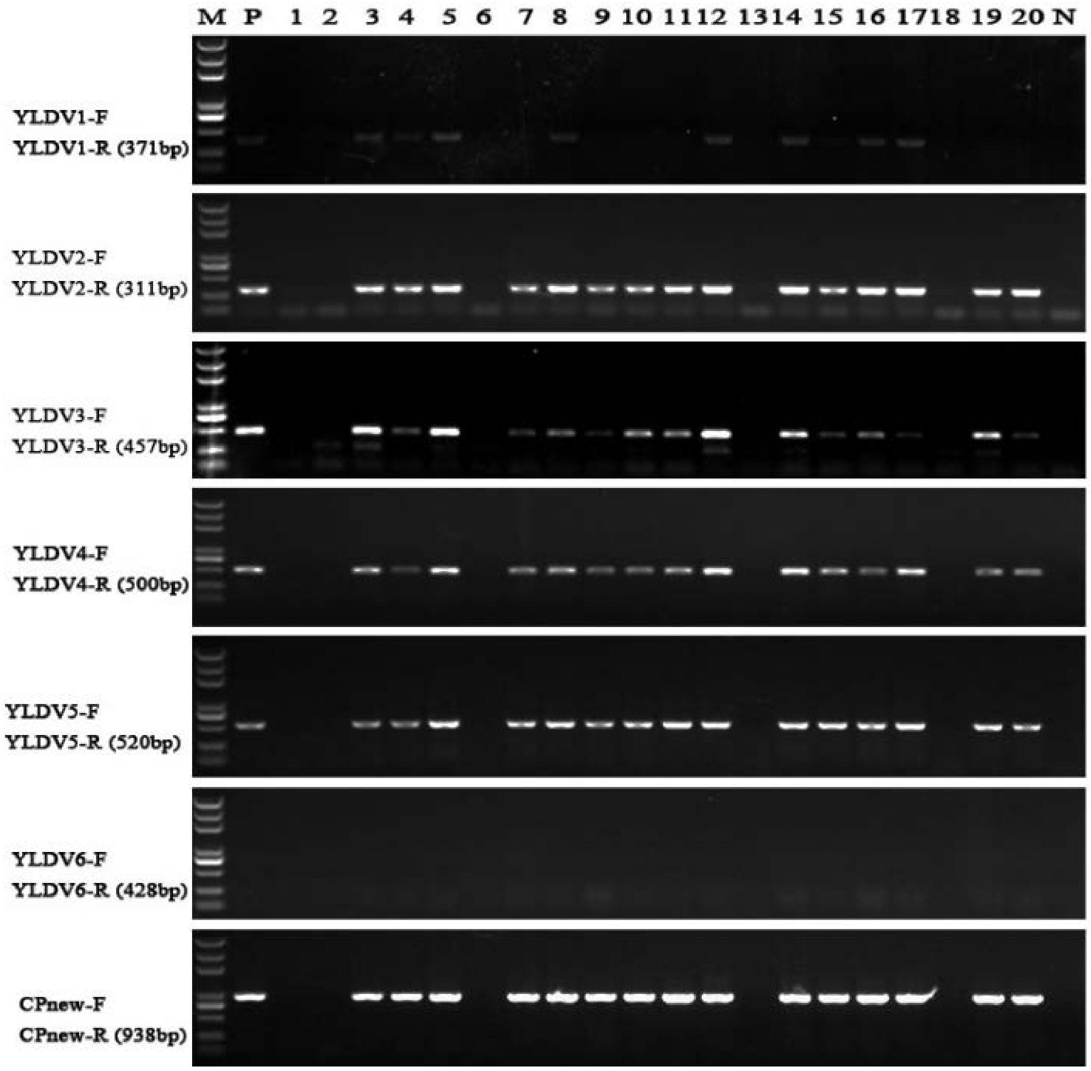

Figure S3. Primers evaluation for APV1 detection. Amplified DNA fragments were examined by electrophoresis on 1% agarose gels and dyed with EtBr. YLDV (1-6) were used in the previous reports, and CPnew-F/R was designed in this study based on the conserved regions of 23 APV1 isolates. Twenty areca palm leaf samples were collected from 6 cities and counties in Hainan Province. M, DNA size 2000 marker; P, positive control; N, negative health control.
